# Gluten-free vegan diet induces decreased LDL and oxidized LDL levels and raised atheroprotective natural antibodies against phosphorylcholine in patients with rheumatoid arthritis: a randomized study

**DOI:** 10.1186/ar2388

**Published:** 2008-03-18

**Authors:** Ann-Charlotte Elkan, Beatrice Sjöberg, Björn Kolsrud, Bo Ringertz, Ingiäld Hafström, Johan Frostegård

**Affiliations:** 1 Rheumatology Unit, Karolinska Institutet at Karolinska University Hospital Huddinge, 141 86 Stockholm, Sweden

## Abstract

**Introduction:**

The purpose of this study was to investigate the effects of vegan diet in patients with rheumatoid arthritis (RA) on blood lipids oxidized low-density lipoprotein (oxLDL) and natural atheroprotective antibodies against phosphorylcholine (anti-PCs).

**Methods:**

Sixty-six patients with active RA were randomly assigned to either a vegan diet free of gluten (38 patients) or a well-balanced non-vegan diet (28 patients) for 1 year. Thirty patients in the vegan group completed more than 3 months on the diet regimen. Blood lipids were analyzed by routine methods, and oxLDL and anti-PCs were analyzed by enzyme-linked immunosorbent assay. Data and serum samples were obtained at baseline and after 3 and 12 months.

**Results:**

Mean ages were 50.0 years for the vegan group and 50.8 years for controls. Gluten-free vegan diet induced lower body mass index (BMI) and low-density lipoprotein (LDL) and higher anti-PC IgM than control diet (*p *< 0.005). In the vegan group, BMI, LDL, and cholesterol decreased after both 3 and 12 months (*p *< 0.01) and oxLDL after 3 months (*p *= 0.021) and trendwise after 12 months (*p *= 0.090). Triglycerides and high-density lipoprotein did not change. IgA anti-PC levels increased after 3 months (*p *= 0.027) and IgM anti-PC levels increased trendwise after 12 months (*p *= 0.057). There was no difference in IgG anti-PC levels. In the control diet group, IgM anti-PC levels decreased both after 3 and 12 months (*p *< 0.01). When separating vegan patients into clinical responders and non-responders at 12 months, the effects on oxLDL and anti-PC IgA were seen only in responders (*p *< 0.05).

**Conclusion:**

A gluten-free vegan diet in RA induces changes that are potentially atheroprotective and anti-inflammatory, including decreased LDL and oxLDL levels and raised anti-PC IgM and IgA levels.

## Introduction

Patients with rheumatoid arthritis (RA) have increased cardiovascular disease (CVD) and mortality [1-3]. Several recent studies indicate an increased prevalence not only of CVD but also of atherosclerosis as determined by ultrasound of carotid arteries [1,4,5]. The underlying mechanisms causing this increased risk are not wholly clarified but inflammation and disease duration are suggested to be of importance [6-9]. Also, extra-articular RA has been described as important in RA-related mortality and CVD [10]. Patients with RA also have a disturbed lipoprotein profile associated with disease activity [11]. The dyslipidemia is often presented with normal or decreased low-density lipoprotein (LDL) cholesterol, low high-density lipoprotein (HDL) cholesterol, and high triglycerides (TGs) in a manner comparable to inflammatory and infectious diseases in general [12]. Treatment with disease-modifying anti-rheumatic drugs has been shown to improve lipid profile in treatment responders but not in non-responders [4] and is inversely correlated to changes of C-reactive protein (CRP) and erythrocyte sedimentation rate (ESR) [13]. These results suggest that the abnormal lipid levels are related to inflammation. On the other hand, treatment with long-term infliximab surprisingly induced a pro-atherogenic profile despite reduced inflammatory activity [14]. Another treatment that might affect the dyslipidemia in RA is dietary manipulation such as potential anti-inflammatory agents. The possibility that such manipulation regulates the disturbed lipoprotein profile, however, has been addressed less in RA. In this respect, vegan diet with a low content of saturated fat and a higher intake of polyunsaturated fat should be of special interest. Oxidized or other types of modified LDL generally are believed to be of importance in atherosclerosis and CVD since such LDL is taken up in the artery wall by macrophages and has immune stimulatory and pro-inflammatory properties [15,16]. We have focused on the role of antibodies against PC (anti-PCs) and recently demonstrated that high anti-PC IgM levels predict a favorable outcome in the development of human atherosclerosis [17]. PC is immunogenic when exposed to the immune system (for example, in infectious agents, including *Streptococcus pneumoniae *and some nematodes). Anti-PCs belong to a group of natural antibodies, which also are present in mice in the absence of exposure to any microorganisms [18]. Earlier, we have shown that a gluten-free vegan diet during 1 year significantly reduced disease activity and decreased levels of antibodies to beta-lacto globulin and gliadin in patients. with RA [19]. In the present study, we addressed the effect of a gluten-free vegan diet on risk factors, including blood lipids, oxidized LDL (oxLDL), and anti-PCs for atherosclerosis.

## Materials and methods

### Patients

Sixty-six patients with RA according to the criteria of the American College of Rheumatology (ACR) [20] were enrolled into the study as previously described [19]. In brief, they were eligible for inclusion if they were between 20 and 69 years of age, had a disease duration of between 2 and 10 years, had not tried dietary manipulation before, and had active disease. Active disease was considered present if the patients fulfilled two of the following three criteria: duration of early morning stiffness of at least 1 hour, ESR of at least 30 mm, and six or more swollen and/or tender joints. Exclusion criteria were current malignancy; severe cardiovascular, pulmonary, or renal disease; and diabetes mellitus. Patients were allowed to continue on non-steroidal anti-inflammatory drugs, oral glucocorticoids, and anti-rheumatic therapy. If necessary, these medications could be changed during the study. Thus, two patients in the vegan group and one in the non-vegan group started anti-rheumatic therapy during the study period. None of the patients used statins or biologic medications before or during the study. All patients received an addition of 1 mg/day of vitamin B_12 _and 50 μg/day of selenium. The patients were randomly assigned to two different diets, either a vegan diet free of gluten or a non-vegan diet for 1 year, using a minimization technique as described [19]. This randomization technique was used to avoid an imbalance in the following variables: age, disease duration, and concomitant anti-rheumatic treatment [19]. The study was approved by the Karolinska University Hospital ethics committee and was performed in accordance with the Declaration of Helsinki. We received informed consent from the patients.

### Study diets

#### Vegan diet free of gluten, called vegan diet

The patients randomly assigned to a vegan diet (n = 38) started with 1-day low-energy fasting, with vegetable broth and berry juices, followed by the gluten-free vegan diet for 1 year. In the vegan diet, protein energy level was 10% of the total energy intake, the carbohydrates 60%, and fat 30%. The vegan diet contained vegetables, root vegetables, nuts, and fruits. As gluten was not permitted, the diet contained buckwheat, millet, corn, rice, and sunflower seeds. Unshelled sesame seeds in the form of sesame milk were a daily source of calcium.

#### Well-balanced diet, called non-vegan diet

In all, 28 patients were randomly assigned to a well-defined non-vegan diet. This diet contained 10% to 15% protein, 55% to 60% carbohydrate, and no more than 30% fat, of which saturated fat was not supposed to make up more than 10% of the total energy intake. This implies a variety of foods from all food groups. Five or more daily servings of fruits and vegetables were recommended as well as increasing intakes of starch and other complex carbohydrates by eating potatoes, bread, and cereals and selecting whole-grain products as often as possible [19].

#### Dietary intake follow-up

The introductory intervention period lasted 6 to 7 days for both groups. During that period, the patients received instructions in the theory and practical preparation of the diet in question. Thereafter, each patient had access to advice and help from one of two physicians, a dietician, and nurses in maintaining the respective diet. Follow-up meetings with the different groups, which focused on providing support and advice from the dietician and the nurse, took place at 3, 6, 9, and 12 months. Dietary compliance was assessed by 3-day food intake records at baseline and at 3, 6, 9, and 12 months. Furthermore, at each visit to the clinic, the patients were questioned about compliance with the study diet. Also, between visits, the nurse made phone calls to the patients and the patients had the opportunity to call the nurse when needed.

### Assessments

Body mass index (BMI) was calculated from weight divided by the square of the height (kg/m^2^). Individuals with BMI values of less than 18.5 kg/m^2 ^were classified as underweight/malnourished, between 18.5 and 24.9 kg/m^2 ^as normal, 25 and 29.9 kg/m^2 ^as overweight, and greater than 30 kg/m^2 ^as obese [21].

#### Disease activity

Disease activity was assessed by the composite index Disease Activity Score in 28 joints (DAS28), which includes number of swollen joints, number of tender joints, patient's assessment of global disease activity, and ESR [22]. At 3 and 12 months, EULAR (European League Against Rheumatism) response criteria were recorded [23]. Good responders were those with a DAS28 improvement of at least 1.2 and an endpoint value of less than 3.2. Moderate responders were patients with either an improvement of at least 1.2, independent of the attending DAS28 value, or an improvement of at least 0.6 in combination with an endpoint DAS28 of less than 5.1. Also, the ACR 20% response was determined for each patient [24].

#### Physical function

The Swedish version of the Stanford Health Assessment Questionnaire (HAQ) was used to measure physical function [25]. The HAQ score ranges from 0 to 3, with a higher score indicating a higher degree of disability.

#### Analytical methods

At baseline, before initiation of the diets, and after 3 months and 1 year, blood samples were drawn in the morning after an overnight fast. The biochemical variables were determined automatically by standard laboratory methods with commercial kits. They included ESR, CRP, hemoglobin, white blood count, serum albumin, total cholesterol, LDL, HDL, and TGs. The lipids were considered pathologic when cholesterol was greater than 5.0 mmol/L, LDL greater than 3.0 mmol/L, TGs greater than 2.0 mmol/L, HDL less than 1.0 mmol/L (for men), and HDL less than 1.2 mmol/L (for women). Serum samples were also stored at -70°C until analyzed for oxLDL and natural anti-PCs.

### Oxidized low-density lipoprotein and antibody determinations

OxLDL was determined by use of a commercial kit (Mercodia AB, Uppsala, Sweden) as described by the manufacturer. Antibodies to PC-bovine serum albumin (BSA) were determined by enzyme-linked immunosorbent assay (ELISA) essentially as described [26]. Briefly, pooled serum from medium- to high-titer individuals was used as an internal standard and was tested on every plate. The plateau of antibody binding was reached with the antigen concentration of 10 μg/mL. An F96 microtiter polysorp plate, therefore, was coated with PC-BSA (10 μg/mL) 50 μL/well in phosphate-buffered saline (PBS). Coated plates were incubated overnight at 4°C. After five washings with PBS, the plates were blocked with 2% BSA-PBS for 2 hours at room temperature and washed as described above. Serum samples were diluted (1:30) in 0.2% BSA-PBS and added at 50 μL/well. Plates were incubated overnight at 4°C and washed as described above. Alkaline phosphatase-conjugated goat anti-human IgM, IgA, or IgG (diluted 1:7,000 in the sample buffer) was added at 100 μL/well and incubated at 4°C overnight. After five washings, color was developed by adding the alkaline phosphatase substrate (*p*-nitrophenyl phosphate) at 100 μL/well and incubating the plates for 60 minutes at room temperature in the dark. The plates were read in an ELISA Multiscan Plus spectrophotometer (EMax; Molecular Devices, Sunnyvale, CA, USA) at 405 nm. All samples were measured in duplicates in a single assay, and the coefficients of variation were below 10% to 15%.

### Statistical analyses

The statistics were computed using StatView software (SAS Institute AB, Stockholm, Sweden). Correlation analysis was performed using simple regression for normally distributed variables, and Spearman correlation analysis for non-normally distributed variables. Skewed continuous variables were logarithmically transformed to attain a normal distribution. Study groups were compared using analysis of variance repeated measurements. Effects within groups were compared using the Student *t *test. The significance level was put at a *p *value of less than 0.05.

## Results

Thirty patients in the vegan group and 28 in the non-vegan group completed at least 3 months on the diet regimen and were included in our analyses. Additionally, 8 patients in the vegan group stopped the diet before completing 12 months. The two diet groups were well balanced in regard to patient characteristics and disease activity (Table 1). Furthermore, there were no differences between groups for the metabolic and lipid variables tested except that HDL was higher at baseline among the vegan group as compared with the non-vegan diet group (*p *= 0.03). Of the patients, 45% had pathologic levels of cholesterol, 47% of LDL, 30% of HDL, and 7% of TGs. When effects of the different diet regimens were compared between groups at 12 months, BMI (F = 6.6, *p *= 0.0024), weight (F = 8.9, *p *= 0.003), and LDL (F = 18.8, *p *< 0.0001) were significantly lower and anti-PC IgM (F = 8.0, *p *= 0.0006) was higher in the vegan diet group whereas the increase in anti-PC IgA and decrease in oxLDL differed only trendwise (F = 2.5, *p *= 0.084 and F = 2.6, *p *= 0.081, respectively). DAS28 was higher in the diet control group than in the vegan group (F = 3.1, *p *= 0.047), whereas HAQ score did not differ significantly.

**Table 1:** Patient characteristics for the two diet groups at baseline

	Vegan diet n = 30	Non-vegan diet n = 28	*P *value
Patients, female/male, n	28/2	24/4	
Age in years, mean (CI)	49.9 (46.6–53.3)	50.8 (46.2–55.5)	0.75
Disease duration in years, mean (CI)	5.0 (4.1–6.0)	5.8 (4.7–6.9)	0.31
Rheumatoid factor-positive, n (%)	25 (83)	21 (75)	
Body mass index, kg/m^2^, mean (CI)	24.1 (22.3–25.9)	23.8 (21.6–26.0)	0.81
Weight, kg, mean (CI)	66.4 (61.7–71,1)	67.9 (61.9–73.7)	0.70
DAS28, mean (CI)	5.3 (5.0–5.7)	5.3 (4.9–5.6)	0.80
HAQ score, mean (CI)	1.4 (1.2–1.5)	1.3 (1.1–1.5)	0.44
Patients on DMARDs, n (%)	26 (87)	26 (93)	
Patients on glucocorticoids, n (%)	18 (60)	9 (32)	
Patients on NSAIDs, n (%)	24 (80)	24 (86)	

*P *values are difference between groups. CI, confidence interval; DAS28, Disease Activity Score in 28 joints; DMARD, disease modifying anti-rheumatic drug; HAQ, Health Assessment Questionnaire; NSAID, non-steroid anti-inflammatory drug.

The vegan group patients reduced their weights from 66.4 (61.7 to 71.1) kg at baseline to 62.2 (58.2 to 66.2) kg at the 12-month visit (*p *< 0.001) and BMI from 24.1 (22.3 to 25.9) to 22.7 (21.3 to 24.2) (*p *< 0.001). The corresponding figures for the non-vegan diet group were 67.8 (61.9 to 73.7) kg to 67.1 (60.8 to 73.5) kg and 23.8 (21.6 to 36.0) to 23.4 (20.9 to 25.9), respectively (both non-significant changes).

In the vegan group, DAS28 and HAQ score were significantly reduced at both 3 and 12 months compared with baseline and CRP at 12 months (Table 2). Among the non-vegan diet group, DAS28 showed a slight but significant decrease at 3 months but not at 12 months, whereas HAQ score and CRP were unchanged over time (Table 2). In the vegan group, 63% were good and moderate responders at 12 months compared with 32% of the non-vegan diet patients. The ACR 20% responses were 37% and 4%, respectively. In the vegan group, total cholesterol, LDL, and the ratio LDL/HDL decreased significantly after both 3 and 12 months, whereas TGs and HDL did not change (Table 2). These changes were seen both in responders and non-responders (data not shown). OxLDL levels decreased significantly after 3 months and trendwise after 12 months (Table 2). When patients were separated into ACR 20% responders and non-responders at 12 months, the decrease in oxLDL was seen only in responders and was significant at both 3 and 12 months (*p *< 0.01).

**Table 2:** Disease activity and lipid variables for the patients who followed the diet regimens for at least 3 months

	Vegan diet patients					Non-vegan diet patients				
	Baseline	3 months	*P *value	12 months	*P *value	Baseline	3 months	*P *value	12 months	*P *value

DAS28^a^	5.3 (5.0–5.7)	4.7 (4.3–5.2)	0.002	4.3 (3.8–4.9)	<0.001	5.3 (4.9–5.6)	5.0 (4.6–5.3)	0.014	5.0 (4.6–5.4)	0.19
HAQ score^a^	1.4 (1.2–1.5)	1.1 (0.9–1.3)	0.010	1.0 (0.8–1.2)	0.001	1.3 (1.1–1.5)	1.2 (1.0–1.4)	0.62	1.2 (1.0–1.4)	0.59
CRP^b^	13 (6–26)	11 (5–29)	0.68	5 (4–20)	0.008	22 (5–32)	10 (5–33)	0.07	12 (4–19)	0.28
Albumin^a^	36 (34–37)	34 (33–35)	0.013	36 (35–38)	0.43	35 (34–37)	37 (35–39)	0.06	37 (35–38)	0.06
Cholesterol^b^	5.2 (4.4–5.7)	4.3 (3.9–5.0)	<0.001	4.3 (3.8–5.1)	0.003	4.7 (4.2–5.3)	5.1 (4.3–5.5)	0.20	5.0 (4.0–5.5)	0.68
Triglycerides^a^	1.1 (1.0–1.3)	1.2 (1.0–1.3)	0.43	1.1 (0.9–1.2)	0.32	1.1 (0.9–1.5)	1.2 (0.7–1.6)	0.79	1.1 (0.8–1.3)	0.69
HDL^a^	1.4 (1.3–1.5)	1.3 (1.2–1.4)	0.14	1.4 (1.3–1.6)	0.22	1.3 (1.2–1.3)	1.3 (1.2–1.4)	0.045	1.3 (1.2–1.4)	0.07
LDL^b^	3.2 (2.6–3.7)	1.3 (1.2–1.6)	<0.001	2.4 (2.1–3.0)	<0.001	2.9 (2.5–3.4)	3.1 (1.2–1.5)	0.23	3.2 (2.4–3.5)	0.83
LDL/HDL^a^	2.7 (2.2–3.1)	2.0 (1.8–2.3)	<0.001	1.9 (1.6–2.2)	<0.001	2.4 (2.2–2.7)	2.5 (2.2–2.7)	1.00	2.2 (2.0–2.5)	0.22
oxLDL^a^	54.7 (46.2–63.2)	49.4 (43.0–55.8)	0.021	48.6 (41.7–56.5)	0.09	54.5 (45.9–63.1)	54.8 (46.2–63.4)	0.88	55.2 (45.6–64.7)	0.57
anti-PC, IgM^a^	778 (706–849)	812 (729–896)	0.39	822 (743–900)	0.057	797 (676–918)	742 (620–864)	<0.001	731 (596–870)	0.003
anti-PC, IgG^a^	861 (761–962)	879 (785–974)	0.28	859 (760–957)	0.92	913 (814–1012)	900 (802–999)	0.46	880 (764–996)	0.47
anti-PC, IgA^a^	837 (588–1087)	938 (640–1234)	0.027	854 (595–1112)	0.26 0.62	798 (567–1030)	772 (539–1005)	0.17	745 (357–1028)	0.42

^a^Mean values (confidence interval 95%). ^b^Median values (25th to 75th percentile). *P *values are differences between baseline and 3 and 12 months, respectively, for each diet group. Anti-PC, antibody against phosphorylcholine; CRP, C-reactive protein; DAS28, Disease Activity Score in 28 joints; HAQ, Health Assessment Questionnaire; HDL, high-density lipoprotein; LDL, low-density lipoprotein; oxLDL, oxidized low-density lipoprotein.

IgM anti-PC levels were raised after 3 and 12 months compared with baseline, but the difference reached a trendwise significance only at 12 months (*p *= 0.057). IgA anti-PC levels were higher at 3 months compared with baseline (*p *= 0.020) but were only non-significantly higher after 12 months. There was no difference in IgG anti-PC levels between any time points during the study. Low levels of anti-PC IgM (below 25% percentile) were more common in the non-vegan group than in the vegan group after 3 months (*p *< 0.038). In the non-vegan diet group, HDL was increased significantly after 3 months (*p *< 0.05) and non-significantly after 12 months, whereas other lipids did not change (Table 2). IgM anti-PC levels decreased significantly both after 3 and 12 months (*p *< 0.001). Other antibodies tested did not differ.

## Discussion

Here, we report that a gluten-free vegan diet in patients with RA induced a decrease in total cholesterol, LDL, and the ratio LDL/HDL whereas TGs and HDL did not change significantly. In contrast, the balanced diet in the control group did not influence lipid values significantly. There is now a large body of evidence indicating that this change of lipid profile is favorable in relation to atherosclerosis and CVD, and this diet therefore is likely to be antiatherogenic also in RA. We also report that both BMI and weight decreased significantly in the vegan diet group, which was not the case in the control group. Cholesterol, LDL, and BMI also differed significantly between groups and not only within the vegan group.

These findings are compatible with previous results of vegetarian/vegan dietary regimens in non-RA subjects which have shown lower blood pressure, lower BMI, and lower incidence of CVD [27-29]. Furthermore, these individuals had lower total cholesterol and lower LDL [30,31]. When matched for BMI, subjects on a vegetarian diet had a body fat percentage similar to that of omnivore subjects [32]. Further evidence for the importance of diets on lipoprotein profile is the low incidence of myocardial infarction in Greenland Eskimos, whose high-fat diet is rich in marine lipids [33,34].

In the vegan group, neither the levels of cholesterol, LDL, and the ratio LDL/HDL nor the changes differed between responders and non-responders (in contrast to oxLDL) nor were there any correlations between these changes and inflammatory activity. Such independence of inflammatory activity is similar to that reported concerning changes of linoleic acid found during vegan and vegetarian diets [35]. This implies that the change in lipid profile was a consequence of the vegan diet and not a result of reduced inflammatory activity and that the change in lipids did not have any impact on disease activity in RA.

In contrast to LDL and cholesterol values, TGs did not change as a consequence of vegan diet. In systemic lupus erythematosus, we have reported that high TGs are characteristic of this rheumatic disease and that TGs are strongly associated with disease activity and inflammation) [36,37]. The dyslipidemia in RA therefore may differ somewhat from the 'lupus pattern of dyslipidemia', in which there appears to be a clearer association with inflammation.

Another finding herein is that levels of circulating oxLDL decreased in the vegan group, which was not the case among controls. OxLDL stimulates endothelial and monocyte adhesiveness [38,39] and is taken up by macrophages in the artery wall, which develop into foam cells [40]. OxLDL itself, or otherwise modified LDL, also has many pro-inflammatory and immune stimulatory properties, including activation of T cells [41] and monocytes/macrophages [39]. Therefore, it is possible that the reduction in oxLDL also could contribute to the decreased disease activity due to its anti-inflammatory properties, a possibility supported by the finding that the reduction of oxLDL was seen only in diet responders.

One important mechanism by which oxLDL promotes immune activation is through platelet-activating factor (PAF), PAF-like lipids, and lysophosphatidylcholine in oxLDL which have PC as one determining epitope [42-44]. Furthermore, oxLDL is taken up by macrophages through scavenger receptors, including CD36, which has PC as ligand [45]. Circulating oxLDL has been reported to be a risk marker for CVD and atherosclerosis [46], and decreased levels of oxLDL thus could contribute to a less atherogenic profile. Little is known about a possible role of oxLDL in promoting the chronic inflammation present in RA, but it is interesting to note that oxLDL is present in foam cells in synovia from patients with RA [47], indicating that oxLDL also could play a role in the pathogenesis of RA.

Antibodies against OxLDL (aOxLDLs) are raised in RA, but their clinical importance for CVD and atherosclerosis has been much discussed [48,49]. Initially, aOxLDLs were reported to be pro-atherogenic, but subsequent studies suggest that at least at an early stage of disease they could in fact be anti-atherogenic [48,50-52]. This latter possibility is supported by animal experiments from several groups in which immunization with oxLDL causes decreased atherosclerosis development. Varying results may also depend on the fact that LDL oxidation is difficult to standardize [15,50].

Instead of aOxLDLs, we have focused on anti-PCs, and one major finding is that anti-PC levels of IgM and IgA subclasses were raised after a vegan diet. Low levels of anti-PC IgM were more common in the control group than among vegans. Anti-PC IgM was also significantly higher in the vegan group as compared with the control diet group, whereas there was only a trendwise increase in anti-PC IgA.

We recently reported that there is an inverse correlation between anti-PC IgM levels and atherosclerosis development in humans [17]. Further to this, low levels of anti-PC IgM predict an increased risk of CVD in a population-based study (Frostegård et al, unpublished observation). Furthermore, animal experiments indicate that administration of anti-PCs ameliorates atherosclerosis development [53]. These changes in anti-PC levels which were induced by a vegan diet thus are likely to be antiatherogenic, although the role of anti-PC IgA in this respect has been less studied. PC is known to be immunogenic and present on important human pathogens like *S. pneumoniae *[18], and apoptotic cells expose this antigen, which is normally cryptic [54].

Earlier, we reported a decrease in serum levels of IgG antibodies to gliadin and β-lactoglobulin in the group of patients who responded positively to the vegan diet but not in other patients [19]. This reduction was suggested to be explained by diminished immune response to exogenous food antigens. In contrast, anti-PC was thus raised after a vegan diet. The cause of this increased response is not clear. However, we recently reported that anti-PC IgM is much lower in a Swedish population than in individuals from New Guinea living a traditional life as horticulturalists [55]. Their food contains much less of dairy products, refined fat, and grain-derived food and much more of fish, vegetables, and roots. We found that, in this population, anti-PC IgM levels were associated with a polyunsaturated fatty acid (FA) dihomo-gama-linolic acid 20:3 n-6, and we hypothesized that exposure to easily oxidized FA (for example, in the gut immune system) could elicit more robust anti-PC IgM and IgA levels in contrast to saturated FAs, which are not oxidized. Further studies are needed to clarify whether such a mechanism could be of importance. Hypothetically, the gut flora could be changed and so too could exposure of PC, another possibility that deserves further studies. Whether gluten plays a role in the effects presented herein remains to be elucidated.

Our preliminary experiments indicate that polyclonal human anti-PCs of the IgM subclass can inhibit uptake of oxLDL in macrophages and also inhibit pro-inflammatory effects mediated by PAF-like lipids generated during LDL oxidation (Frostegård et al. unpublished observation). Some of these effects thus could be anti-inflammatory, at least in the context of chronic inflammation. In principle, anti-PCs could play a role also in chronic inflammation as protective antibodies in general as in RA and be developed into novel treatment modalities in addition to other protective antibodies discussed in RA [56].

The vegan group also showed significantly lower levels of CRP, a physiologic marker of subclinical inflammation, which has been shown to be associated with insulin resistance and CVD [57,58]. Elevated levels of CRP have been suggested to reflect overproduction by expanded adipose tissue mass [57]. In line with our previous findings [19], both DAS28 and HAQ decreased in the vegan group whereas only DAS28 at 3 months decreased in the control diet group.

Some limitations in the present study should be considered. First, a small number of patients participated. Nonetheless, the size of our groups was sufficient to detect several differences between diet groups, but we could not exclude the possibility of even more differences if a larger number had been studied. Second, a long-term diet study always poses special questions concerning compliance. The fact that compliance was monitored both via regular contacts between the patients and staff of the project and via dietary intake records made us confident that compliance to diet was high among the patients in both the vegan and non-vegan groups. The change in anti-rheumatic medication was considered too limited to have any consequences for results.

## Conclusion

A vegan diet in RA induced decreased LDL and oxLDL levels and raised levels of natural antibodies of IgA and IgM subclasses to PC. We hypothesize that these changes are atheroprotective since LDL is atherogenic and oxLDL has immune-stimulatory and pro-inflammatory effects in atherosclerosis. Furthermore, anti-PC levels are negatively associated with the development of atherosclerosis. To further clarify which components of a vegan diet and which underlying mechanisms that contribute to the effects described here is therefore of interest both in the context of CVD in RA, and in RA in general, where diet intervention as here has an ameliorating effects in many patients.

## Abbreviations

ACR = American College of Rheumatology; anti-PC = antibody against phosphorylcholine; aOxLDL = antibody against oxidized low-density lipoprotein; BMI = body mass index; BSA = bovine serum albumin; CRP = C-reactive protein; CVD = cardiovascular disease; DAS28 = Disease Activity Score in 28 joints; ELISA = enzyme-linked immunosorbent assay; ESR = erythrocyte sedimentation rate; FA = fatty acid; HAQ = Health Assessment Questionnaire; HDL = high-density lipoprotein; LDL = low-density lipoprotein; oxLDL = oxidized low-density lipoprotein; PAF = platelet-activating factor; PBS = phosphate-buffered saline; PC = phosphorylcholine; RA = rheumatoid arthritis; TG = triglyceride.

## Competing interests

JF has received reimbursements from and holds shares in Athera Biotechnologies AB (Stockholm, Sweden). One focus of this company is a biotech company that develops antibody assays, including against PC. JF is named as coinventor on some patents relating to anti-PC. The other authors declare that they have no competing interests.

## Authors' contributions

A-CE participated in data analysis and preparation of the manuscript. BS carried out the oxLDL assay and participated in data analysis and preparation of the manuscript. BK carried out the anti-PC ELISA and participated in data analysis and, to some extent, in preparation of the manuscript. BR participated in the study design. IH participated in data analysis and preparation of the manuscript and designed the vegan study. JF had the main responsibility for data analysis, preparation of manuscript, and hypotheses regarding the roles of oxLDL and anti-PC. All authors read and approved the final manuscript.
